# Uric Acid Solvents in Gout and Gravel

**Published:** 1893-11-25

**Authors:** 


### NEW DRUGS AND PREPARATIONS.

[We shall be glad to receive, at our Office, 428, Strand, London, W.C., from the manufacturers, specimens of all new preparations and drugs which may be brought out from time to time.]

### URIC ACID SOLVENTS IN GOUT AND GRAVEL.—(*Concluded.*)

A final question presents itself. Are the uric acid solvents of any value in either of the diseases which we have been considering? We reply that if their compounds with uric acid decompose in the urine more slowly than the ordinary urates do into uric acid and biurates, or if the existing renal calculi can be reduced or dissolved by them, or if we wish for local applications to diseased joints and concretions in gout, then there is plenty of scope for the most powerful solvents we can find. In patients, too, who have recovered from a gouty state, where the percentage of urates in the blood has fallen very low, and there is no longer any risk of uratosis, the deposits around joints and the concretions are gradually removed by the serum. Here, again, in the restoration of the crippled joints, solvents given internally may have great value, like blisters and gentle massage externally, though any action in such tissues must be extremely slow. They cannot prevent the paroxysm, but they may remove its effects.

As to the choice of these agents, all that can be said is that piperazine has seven times greater solvent power than lithia in test tube experiments, and a case is recorded where in renal colic an immense quantity of gravel was passed with much relief from pain as soon as piperazine began to be given. Stewart has also recorded instances of very great benefit in renal calculus, and were it not so expensive, there is little doubt of its being widely used, especially in cases unsuitable for surgical treatment, or in the early stages of calculi, where it may remove the entire concretions. A gout water containing 15 grains each of phenocoll and piperazine is now highly praised in Germany. A still newer solvent is Edison's tetra ethylammonium, given in 5 to 20 minim doses of a 10 per cent. solution three times a day. It should be diluted freely, since it is very bitter and strongly alkaline. Its solvent power on uric acid is probably greater and more rapid than that of any other substance. Mr. Edison was seeking for a substance which might be applied on positive galvanic electrodes to gouty joints and concretions, but this ethyl can be given internally as well as locally without harm. As yet there is hardly any clinical experience of its powers, but no objectionable symptoms have been so far caused by its use. We must be careful to distinguish it from the methyl ammonium, which is a powerful poison.

Solvents for uric acid are of value then, not as preventing uratosis, but as aids in removing its effects, either locally or internally, when the blood is no longer saturated with biurates. They are of use in the early stages of renal calculi, and possibly in preventing them. We have not yet evidence to say whether any of them increase the elimination of uric acid salts in gout, but it may be that such a thing is impossible unless we could transplant an extra kidney or two into each gouty patient.

